# A Simple and Inexpensive Electrochemical Assay for the Identification of Nitrogen Containing Explosives in the Field

**DOI:** 10.3390/s17081769

**Published:** 2017-08-02

**Authors:** Jeffrey S. Erickson, Lisa C. Shriver-Lake, Daniel Zabetakis, David A. Stenger, Scott A. Trammell

**Affiliations:** Center for Biomolecular Science and Engineering, Naval Research Laboratory, Washington, DC 20375, USA; jeffrey.erickson@nrl.navy.mil (J.S.E.); lisa.shriverlake@nrl.navy.mil (L.C.S.-L.); daniel.zabetakis@nrl.navy.mil (D.Z.); david.stenger@nrl.navy.mil (D.A.S.)

**Keywords:** electroanalytical chemistry, explosives detection, field assay

## Abstract

We report a simple and inexpensive electrochemical assay using a custom built hand-held potentiostat for the identification of explosives. The assay is based on a wipe test and is specifically designed for use in the field. The prototype instrument designed to run the assay is capable of performing time-resolved electrochemical measurements including cyclic square wave voltammetry using an embedded microcontroller with parts costing roughly $250 USD. We generated an example library of cyclic square wave voltammograms of 12 compounds including 10 nitroaromatics, a nitramine (RDX), and a nitrate ester (nitroglycine), and designed a simple discrimination algorithm based on this library data for identification.

## 1. Introduction

There is a need for lightweight and inexpensive electrochemical sensors for the identification of explosives in the field. Currently, hand-held technologies deployed for field detection and identification have a number of important limitations [1,2,3]. Hand-held systems for spectroscopic identification including IR spectroscopy and Raman spectroscopy are not well suited to detecting compounds at trace levels [3,4]. Colorimetric devices have been developed but are prone to false positives and report only classes of explosives using costly consumables [1,4]. Ion mobility spectrometry systems identify explosives and are handheld but rely on a radioactive source such as nickel-63 or americium-241 [1,3,4]. Amplifying fluorescent polymers such as the Fido X3 provides broad-band but non-specific trace explosive detection [1,3]. Other techniques such as mass spectrometry and gas chromatography tend to be time-consuming assays and are bulky [1,4].

Electroanalytical techniques are useful for the detection of explosives because the instrumentation is simple and can support a wide variety of assays [5,6,7,8,9,10,11,12,13,14,15,16]. Recent advances include solid-state sensors for forensics [17], ionic liquid gel-polymer electrolytes for disposable electrodes [18], and advanced chemometric data treatment for identification [19,20]. There has also been a trend towards custom built potentiostats [21,22,23,24,25]. These instruments boast several advantages including small size and very low cost, and are frequently battery operated. Perhaps the most well-known instrument in this family is the CheapStat, which was developed for general analytical and educational purposes [26]. From this report, we realized that a much more specialized instrument could be developed. It would retain the advantages of being inexpensive, miniaturized, and low power, but would be tailored specifically for the detection of explosives. Furthermore, it would be suitable for both handheld use and for integration into autonomous unmanned vehicles.

In this is work, we report the development and testing of an electrochemical-based solution for the detection of explosives in the field: a simple wipe-test assay run by a hand-held prototype single-board adder potentiostat of the type described by Bard [27] and commonly used in benchtop commercial instruments. The prototype system is capable of performing highly sensitive time-resolved electrochemical measurements with parts costing $250 USD. Electrochemical parameters needed for the detection of explosives are discussed, keeping in mind the overall goal of minimal cost, size and power. An example library of square wave voltammograms with a simple discrimination algorithm for identification of nitrogen containing explosives is presented.

## 2. Materials and Methods

### 2.1. Materials

Analytical explosive standards (1 mg/mL in CH_3_CN) were purchased from Sigma-Aldrich and included 2,4,6-trinitrotoluene (TNT), 1,3,5-trinitrobenzene (TNB), 2,4,6-Trinitrophenylmethylnitramine (Tetryl), 2,4-Dinitrotoluene (2,4-DNT), 2,6-Dinitrotoluene (2,6-DNT), 1,3-Dinitrobenzene (1,3-DNB) 4-amino-2,6-dinitrotoluene (4-am-2,6-DNT), 2-amino-4,6-dinitrotoluene (2-am-4,6-DNT), 2-nitrotoluene, (2-NT), 3-nitrotoluene, (3-NT), 1,3,5-Trinitroperhydro-1,3,5-triazine (RDX) and Propane-1,2,3-triyl trinitrate (NG). Screen printed electrodes (SPEs) were purchased from Pine Research with a carbon working electrode with dimensions of 4 mm × 5 mm. A 50 mM potassium phosphate buffer was prepared from K_2_HPO_4_ and adjusted to pH 6.5 with concentrated HCl.

### 2.2. Prototype Instrument Construction

Our prototype instrument is a heavily modified version of a previously reported open-source potentiostat, the CheapStat [26]. It was designed and built in a similar manner. Briefly, we started by evaluating the CheapStat and identifying features that needed to be added or improved. A new circuit design was created in Eagle 7 (Autodesk Inc., San Rafel, CA, USA) and a layout file was developed. These designs were sent to Royal Circuit Solutions (Hollister, CA, USA) for fabrication. Electronic components were purchased from Mouser Electronics (Mansfield, TX, USA). Assembly of the bare circuit boards was performed in house with convective rework tools. The firmware used to run the embedded microcontroller was written in C Programming Language using the CodeVision AVR compiler (HP Info Tech, Bucharest, Romania). A simple graphical user interface (GUI) was developed to run the instrument. It was also programmed in C Programming Language using the LabWindows/CVI compiler (National Instruments, Austin, TX, USA). A simple battery mount was machined out of 1/4” high density polyethylene (HDPE) plastic and the entire device was assembled using aluminum standoffs.

The prototype instrument was designed with autonomous operation in mind. The software GUI is used to send instructions to the instrument and to receive and process data after scans are complete, but need not be in contact with the instrument while scans are being executed. The prototype instrument contains 1 MB of flash memory, which is sufficient to store roughly 45 min of continuous instrument data before it needs to report back to the GUI. The firmware can be instructed to run up to 32 consecutive scans of different types, with user-specified delays between scans if desired. A built-in quartz crystal oscillator allows for accurate timing between and during scans. As part of the experimental setup, communications are easily programmed so that the instrument reports data at user-specified intervals: this can be live reporting every 5 s, reports at the end of each scan, reports at the end of selected scans, a full report at the end of the entire run, or no reports at all. Full data downloads can also be requested while the instrument is idle.

For ease of data transfer, nearly all of the overhead required to run scans was built directly into the firmware. Only a few bytes of data are required to specify each scan. Three different types of experiments were pre-programmed into firmware (constant potential, cyclic voltammetry, and square wave voltammetry), although the addition of other types of scans is straightforward.

### 2.3. Explosive Electrochemistry and Library Development

The library samples were prepared by evaporating 20 µL (50 µL was used for 3-NT due to low signal strength) of the analytical standards (1 mg/mL in CH_3_CN) on a 1 cm^2^ filter paper placed on Teflon. After being air dried at room temperature, the filter paper was placed on top of a fresh SPE with 100 µL of 50 mM potassium phosphate buffered at pH 6.5 and measured. Test wipe samples were created by the evaporation of 10 µL of the analytical standards on the bench top surface and tested by a simple wipe using filter paper wetted with acetone and then were placed on top of a fresh SPE with 100 µL of 50 mM potassium phosphate buffer. The cyclic square wave voltammograms were recorded at a frequency =17 Hz and an amplitude of 25 mV with a current range =2 mA between 1.2 V and −1.8 V vs. Ag/AgCl with two 1 min accumulation steps at 1.2 V and −1.8 V. All peak potentials (E_p_) are reported as V vs. Ag/AgCl. Sample measurements were operated with a single button preprogramed with the cyclic square wave voltammogram parameters. Data was transferred to the laptop computer by Bluetooth wireless for analysis.

## 3. Results and Discussion

Our primary goal was to develop an assay, a library-based identification algorithm, and a prototype instrument capable of detecting explosives in the field. The instrument must be inexpensive, miniaturized, battery operated, and suitable for integration as a component into both handheld devices and autonomous unmanned systems. Rather than relying on the predefined parameters of a proprietary commercial unit, we chose to work with an open-source potentiostat. In this way, critical variables could be optimized by designing the instrument to have just enough features to complete the required tasks, while leaving room to add additional capabilities in the future such as motor drivers for unattended sampling.

### 3.1. Prototype Instrument

Recently, an open source hand held instrument called the CheapStat [26] was reported. Unfortunately, experiments revealed that the CheapStat was not suitable for our purpose. The voltage sweep range was too small to capture the reduction of the explosives and the oxidation of the reduction products, the compliance voltage was too low, and its single range resistor design could not capture enough dynamic range. Building off the CheapStat concept an increase to the open source potentiostat’s operational parameters was desired for use as a field instrument.

**Figure 1:**
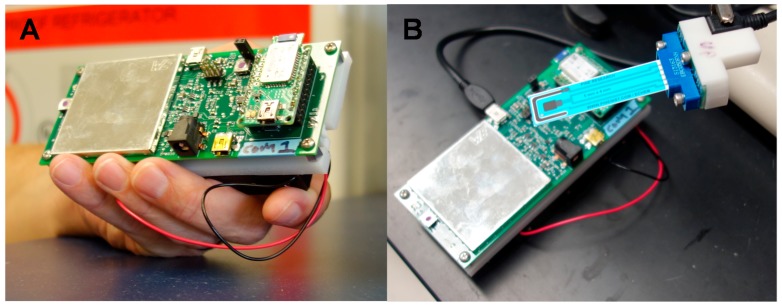
(**A**) The prototype instrument is 2.2” × 5.5” × 0.5” and weighs 300 g with batteries. (**B**) A standard (shielded) USB cable can be used to connect the instrument to a commercially available disposable electrode and socket. Breakout cables may also be used.

The prototype instrument circuit board has a 2.2” × 5.5” × 0.5” footprint and weighs less than 300 g with batteries and less than 60 g without. The instrument sweeps in steps of 1 mV and has a dataset accurate to 16 bits. The unit has two options for power: six AA-size rechargeable NiMH batteries (for handheld operation, endurance is roughly 20 h at a 100% duty with batteries rated at 2000 mAh) or a DC barrel jack (to directly interface with an unmanned vehicle or robot). Maximum continuous power consumption is less than 1 W. This can be reduced by an additional 25% by turning off the wireless communications. The instrument can be manually or automatically switched between six gain ranges with maximum current measurements from ±200 nA to ±20 mA and can sweep a 4 V range between +2 and −2 V. The compliance voltage is 6.1 V. Pictures of the instrument are shown in Figure 1.

The instrument contains a tethered USB communications option that can be used to interface directly with an unmanned vehicle or laptop computer. It also has Class 1 Bluetooth wireless with a 100 m range, which is especially important for remote operation to protect the end user in dangerous situations. Sample measurement can be initiated by pressing a single button on the instrument, or can be started remotely if user safety is an issue. Several electroanalytical techniques have been programmed including cyclic voltammetry, square wave voltammetry, and cyclic square wave voltammetry with accumulation steps at each end of the potential sweep. The measured data is sent back to a remote laptop or cell phone where it can be analyzed automatically using a simple algorithm based on Excel for the explosive identification.

### 3.2. Electrochemical Library

**Figure 2:**
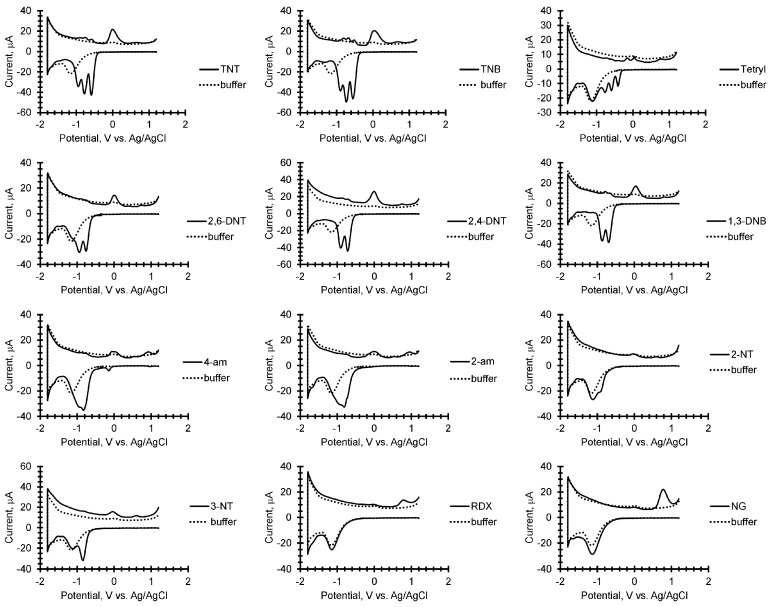
Cyclic square wave voltammetry (CSW) library. The CSWs were recorded at a frequency =17.5 Hz, amplitude =25 mV, current range =2 mA, and potential range =1.2 V to −1.8 V vs. Ag/AgCl with two 1-min accumulation steps at 1.2 V and −1.8 V.

Using the prototype instrument, an example library was compiled of cyclic square wave voltammograms of 12 compounds including 10 nitroaromatics, a nitramine (RDX), and a nitrate ester (nitroglycine) with a simple discrimination algorithm for identification. The electrochemistry at carbon electrodes of nitroaromatics, nitramines, and nitrate esters using cyclic and square wave voltammetry has been extensively studied [16]. The electrochemical reduction of explosives at electrode surfaces generates unique electrochemical signatures that can be used for identification. Nitrogen-containing energetic compounds like TNT, DNT, RDX, and NG give square wave voltammograms with a number of peaks, their position and ratios distinctive to their chemical structure, and can be used to develop a library against which unknown samples can be compared. Figure 2 shows a sample library of nitrogen containing explosives generated by our prototype instrument. These are cyclic square wave voltammograms in which there are 1 min accumulation steps at 1.2 V vs. Ag/AgCl and then at −1.8 V vs. Ag/AgCl. The accumulation step at positive voltages increases the amount of explosive at the electrode for detection [16]. At the negative voltage, a product is generated and accumulated at the electrode which also gives a unique signature characteristic of the chemical structure of the product [16]. Each explosive in the library is the result of an average of three cyclic square wave voltammograms.

For nitroaromatics, the reduction proceeds with the sequential reduction of the nitro groups generating a square-wave voltammogram with the numbers of peaks corresponding to the number of nitro groups in the structure. For all of the nitrogen explosives tested in this study, the cyclic square wave voltammograms are shown in Figure 2 and the cathodic and anodic peak potentials are listed in Table 1. For example 2-TNT, TNB, and Tetryl have three well resolved cathodic peaks. Also, 2,4-DNT, 2,6-DNT, and 1,3-DNB have two well resolved peaks while 2-am-4,6-DNT and 4-am-2,6-DNT have two peaks overlapping. Finally, 2-NT and 3-NT have one reduction peak each. The position of the peaks varies depending on the structure of the compound, i.e., the number nitro groups, amine groups, benzene, or toluene rings with Tetryl having the lowest first reduction peak at −0.41 V and 2-NT the highest reduction peak at −0.92 V. In contrast, the square-wave voltammograms of both RDX and NG show only one broad reduction peak occurring at significantly more negative voltages.

In the reverse sweep, the oxidation of the reduction product is also measured when using cyclic square wave voltammetry and becomes more pronounced with an accumulation step. For nitroaromatics, the reduction product has a major oxidation peak at 0.004 V with a shoulder at 0.09 V for TNT. For TNB the two peaks become more resolved at 0.04 V and 0.011 V. For Tetryl the two oxidation peaks become distinct at −0.15 V and 0.031 V with an additional peak at 0.78 V becoming prevalent.

For 2,6-DNT, 2,4-DNT, and 1,3-DNB the oxidation peaks of the reduction product are at 0.00 V, −0.01 V, and 0.04 V, respectively. For 4-am the oxidation peak is broad and centered at 0.0V. For 2-am, the oxidation peaks are split at −0.076 V and 0.02 V. For the single nitrotoluene compounds, these oxidation peaks are less pronounced and are at −0.02 V and −0.60 V for 2-NT and −0.03 V and −0.60 V for 3-NT. For RDX and NG the major oxidation peaks for the reduction product occurs at 0.80 V and 0.78 V respectively.

Unlike the cathodic sweep, there are also a number of minor peaks observed in the reverse anodic sweep initiated with an accumulation period for the nitroaromatic compounds. In our study, the minor peaks are very clear with TNT, TNB, Teryl, 4 am-2,6-DNT, and 2 am-4,6-DNT, but less prevalent with 2,4-DNT, 2,6-DNT, 2-NT, and 3-NT under the conditions tested and are italicized as peaks 1–3 under the anodic peak potentials in Table 1 footnoted as minor peaks.

### 3.3. Sample Identification

A simple discrimination algorithm was developed in Excel for explosives identification for use in conjunction with our prototype instrument with analysis performed between 1.2 and −1.5 V vs. Ag/AgCl. The algorithm has two steps; the first is a calculation using Equation (1) in which the voltammograms from the library of explosives are used to match the voltammogram of a measured sample by means of a least squares fitting routine using Solver in Excel [28]. In Equation (1), *x_i_* is the current at the *i*th potential in the sample voltammogram and *r_ij_* is the corresponding current value at the same potential for a *j*th reference voltammogram from the library. A coefficient for each *j*th reference (a*_j_* for the cathodic sweep and b*_j_* for the anodic sweep) is multiplied to each current point of the reference voltammogram and used iteratively to fit the measured sample by minimizing the sum of the least squares difference in Equation (1) between the sample and generated fit from library. Both cathodic and anodic sweeps are calculated separately and then summed to get the total coefficient, i.e., a*_j_* + b*_j_* = A*_j_*, which represents the weighted library reference voltammograms used in the calculated fit to match the sample voltammogram.

(1a) $$Sum\;of\;least\;squares=\;\sum^{\text{​}}\;{\left(x_{i}-\;\sum^{\text{​}}a_{j}r_{ij}\right)}^{2};\;\mathrm{cathodic}\;\mathrm{sweep}$$

(1b) $$Sum\;of\;least\;squares=\;\sum^{\text{​}}\;{\left(x_{i}-\;\sum^{\text{​}}b_{j}r_{ij}\right)}^{2}.\;\mathrm{anodic}\;\mathrm{sweep}$$

The second step is a calculation using a correlation function (Equation (2)) that has found some utility in spectral library searching in IR and Raman spectroscopy [29]. We have also found that the correlation calculation is suited for this work in voltammogram library searching. In Equation (2), the first derivative is taken as $y_{i}=\left(x_{i}-x_{i-1}\right)/\Delta$ and $s_{ij}=(r_{ij}-r_{ij-1})/\Delta$ (where Δ = 1 mV) to correct for any baseline variation.

(2) $$c_{j}=\frac{\left[\left(\sum^{\text{​}}y_{i}\times s_{ij}\right)\times \left(\sum^{\text{​}}y_{i}\times s_{ij}\right)\right]}{\left[\left(\sum^{\text{​}}y_{i}\times y_{i}\right)\times \left(\sum^{\text{​}}s_{ij}\times s_{ij}\right)\right]}.$$

The “overall” score for each reference voltammogram in the library is then computed in Equation (3) as the product of the normalized coefficients from Equation (1) and the correlation from Equation (2).

(3) $$score=c_{j}\times \frac{A_{j}}{\sum^{\text{​}}A_{j}}.$$

**Figure 3:**
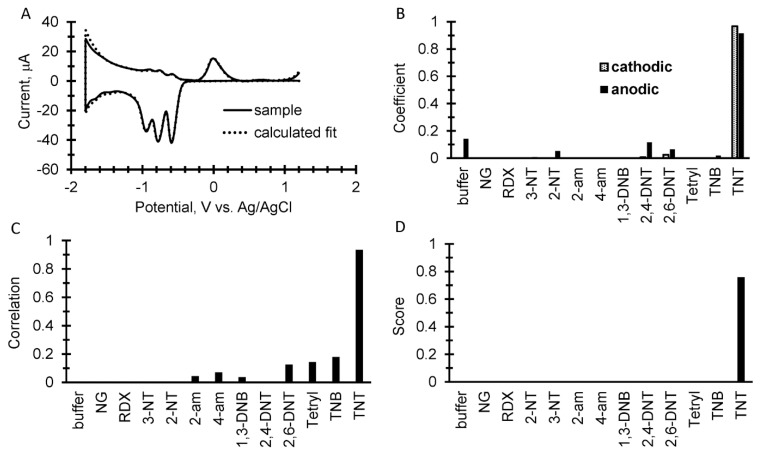
A simple discrimination algorithm for identification. The example is 20 µg of TNT spotted onto filter paper. (**A**) An overlay of a measured cyclic square wave voltammogram with the calculated fits of both the anodic and cathodic sweeps using coefficients from the explosive’s library. Both sample and library voltammograms are zeroed at 0.5 V vs. Ag/AgCl. (**B**) A plot of the resulting coefficients from the fitting calculation for the result shown in A. (**C**) The correlation calculation comparing the measured sample to the square wave voltammetry library. (**D**) The score displayed as the product of the coefficients and correlations.

A representative example of our simple discrimination algorithm is shown in Figure 3 for TNT in which 20 µg of TNT was spotted on a 1 cm^2^ filter paper and place in 100 µL of buffer on the screen printed electrode. The prototype instrument was pre-programed to record a cyclic square wave voltammogram at 17 Hz between 1.2 and −1.8 V vs. Ag/AgCl at a 2 mA current range with a 1 min accumulation step at each positive and negative end of the cycle. At the end of the measurement, the instrument sent the data to a laptop using a Bluetooth connection in which the simple discrimination algorithm in Excel analyzed the data and generated a score. For the analysis, both the sample and library voltammograms are zeroed at 0.5 V vs. Ag/AgCl. Step one of the algorithm is shown in Figure 3A in which a sample voltammogram is overlaid with the calculated fit from the library. Figure 3B shows the resulting coefficients from the fit used in the library and Figure 3C is the correlation calculation from Equation (2). Figure 3D is the score from each component of the library calculated as the product of the correlations and normalized coefficients from the analysis.

**Figure 4:**
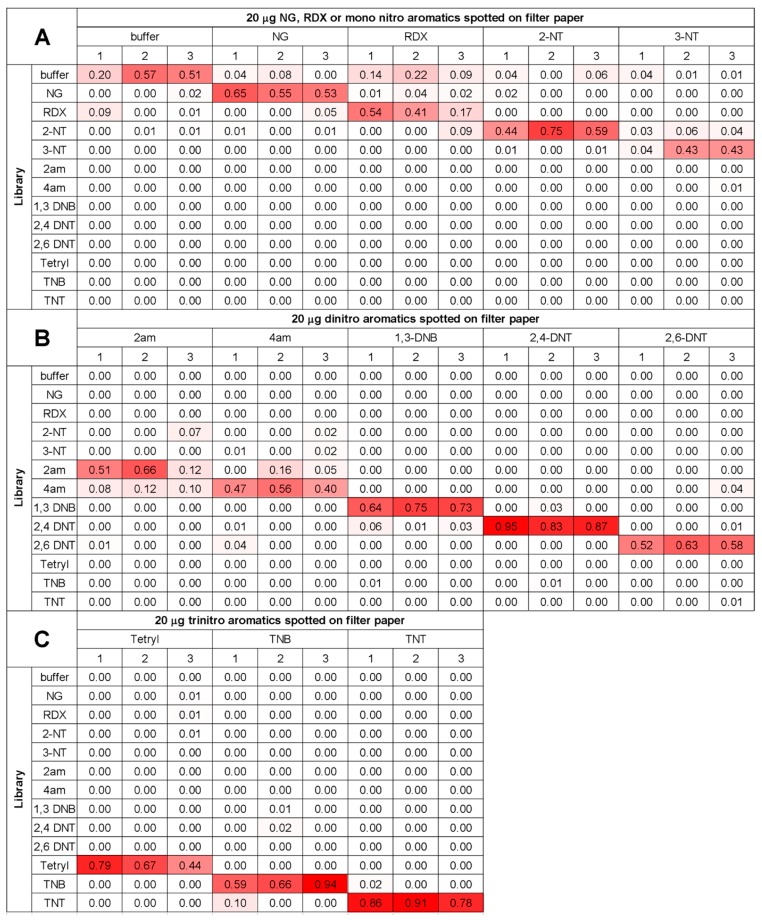
Score charts of each explosive compared to the library. (**A**) 20 µg of NG, RDX, or NT spotted onto filter paper. (**B**) 20 µg of di-nitroaromatics spotted onto filter paper. (**C**) 20 µg of tri-nitroaromatics spotted onto filter paper.

To evaluate that library and simple discrimination algorithm, the library for all of the explosive samples were compared and tested in triplicate under the same conditions. Figure 4 shows the scores all the explosive tested against the library arranged in three categories. One clear observation from the scoring is that explosives with more peaks and stronger signals give higher scores than with explosives that give poorer signals with fewer peaks. For example in the first category, the buffer, NG, RDX, and NT represents the sample signatures having the fewest features and as a result gave overall smaller scores. Categories two and three, i.e., the di- and tri-nitroaromatics have more features in their signatures resulting in higher scores.

### 3.4. Dry Surface Sampling

**Figure 5:**
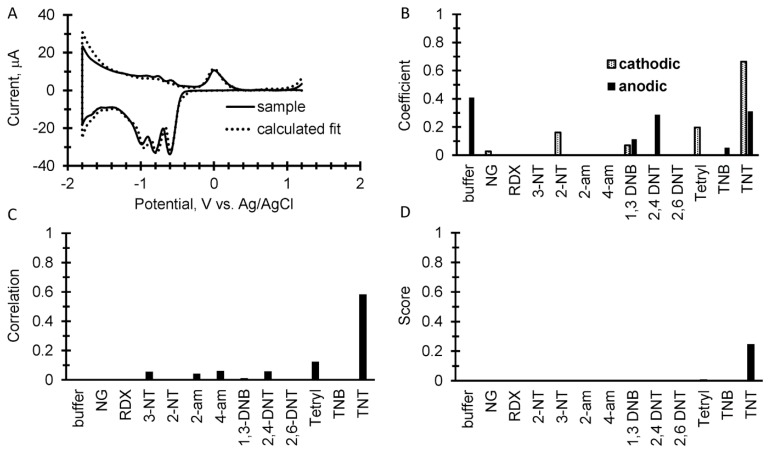
10 µg of TNT wiped from a bench top. (**A**) An overlay of the background subtracted cyclic square wave voltammogram with the calculated fits of both the anodic and cathodic sweeps using coefficients from the explosive’s library. (**B**) A plot of the resulting coefficients from the fitting calculation for the result shown in A. (**C**) The correlation calculation comparing the measured sample to the square wave voltammetry library. (**D**) The score displayed as the product of the coefficients and correlations.

**Figure 6:**
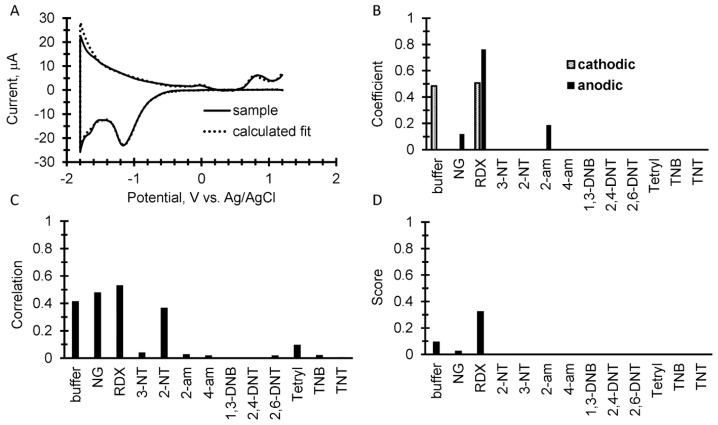
10 µg of RDX wiped from a bench top. (**A**) An overlay of the background subtracted cyclic square wave voltammogram with the calculated fits of both the anodic and cathodic sweeps using coefficients from the explosive’s library. (**B**) A plot of the resulting coefficients from the fitting calculation for the result shown in A. (**C**) The correlation calculation comparing the measured sample to the square wave voltammetry library. (**D**) The score displayed as the product of the coefficients and correlations.

**Figure 7:**
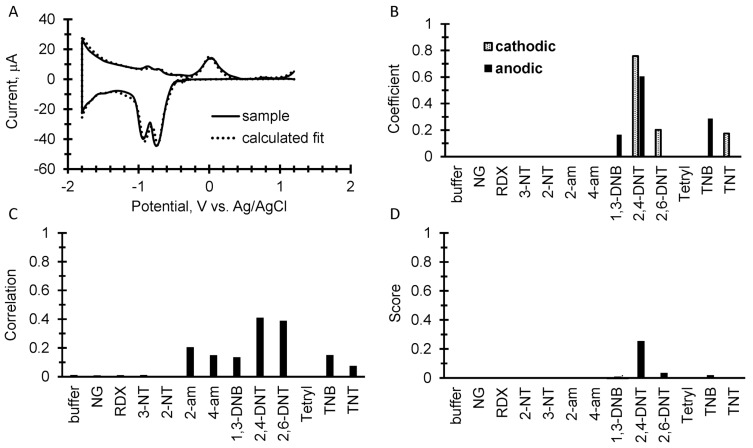
10 µg of 2,4 DNT wiped from a bench top. (**A**) An overlay of the background subtracted cyclic square wave voltammogram with the calculated fits of both the anodic and cathodic sweeps using coefficients from the explosive’s library. (**B**) A plot of the resulting coefficients from the fitting calculation for the result shown in A. (**C**) The correlation calculation comparing the measured sample to the square wave voltammetry library. (**D**) The score displayed as the product of the coefficients and correlations.

To further test the library and simple discrimination algorithm, dry surface sampling was performed in which 10 µL of the explosive analytical standards were pipetted onto the bench top and allowed to evaporate. The samples were then wiped up with an acetone-wetted filter paper and then were placed on top of a fresh SPE with 100 µL of 50 mM potassium phosphate buffer. Figure 5, Figure 6 and Figure 7 show results from the wipe tests. In Figure 5B, Figure 6B and Figure 7B, the calculations from Equation (1) show several false positives from the algorithm’s attempt to fit the noise in the sample voltammogram. In addition, the results of Equation (2) as shown in Figure 5C, Figure 6C and Figure 7C calculates a different set of values from the library analysis, along with several other false positives. These false positives from the two different calculations can be minimized and sometimes zeroed out from the product of the two results in Equation (3) to give a more refined score as shown in Figure 5D, Figure 6D and Figure 7D. While the “score” from Equation (3) can be somewhat empirical, in each case the highest score correctly identified the explosive and shows that trace analysis from a wipe test is feasible at the amount tested. However, the assay does have a limit of detection and quantification below this limit will make identification impossible. Those limits will be addressed in a subsequent paper.

## 4. Conclusions

An inexpensive and miniaturized prototype instrument and assay have been specifically designed to identify explosives in the field. An example library of cyclic square wave voltammograms with a simple discrimination algorithm for identification of nitrogen containing explosives has been demonstrated. Development is continuing on a more robust discrimination algorithm that will be analyzed under field conditions, characterizing explosive mixtures, dynamic range, and limits of identification in a forth coming paper highlighting our instrument.

## Figures and Tables

**Table 1:** Peak potentials measured by Cyclic Square Wave Voltammetry (CSW) ^1^ for 12 explosives.

Explosive	E_pc_, (V) vs. Ag/AgCl				E_pa_, (V) vs. Ag/AgCl					
	Peak 1	Peak 2	Peak 3	Peak 4	Peak 1	Peak 2	Peak 3	Peak 4	Peak 5	Peak 6
TNT	−0.59	−0.78	−0.95	-	−0.90 ^2^	−0.75 ^2^	−0.58 ^2^	0.004	0.09 ^3^	0.65 ^2^
TNB	−0.55	−0.73	−0.90	−1.34 ^2^	−0.90 ^2^	−0.73 ^2^	−0.55 ^2^	0.04	0.11 ^3^	0.70 ^2^
Teryl	−0.41	−0.59	−0.77	−1.1	−0.71 ^2^	−0.58 ^2^	−0.40 ^2^	−0.15	0.031	0.78
2,6 DNT	−0.76	−0.94	-	-	−0.9 ^2^	−0.75 ^2^	0.00	-	-	-
2,4-DNT	−0.72	−0.91	-	-	−0.85 ^2^	−0.70 ^2^	−0.01	-	-	-
1,3-DNB	−0.69	−0.87	-	-	−0.78 ^2^	−0.65 ^2^	0.04 ^4^	-	-	-
4-am	−0.78 ^3^	−0.83	−0.92	-	−0.88 ^2^	−0.75 ^2^	−0.076	0.02	0.51 ^2^	0.94
2-am	−0.73	−0.80	−0.94	-	−0.88 ^2^	−0.73 ^2^	−0.18 ^2,3^	0.0 ^3^	0.52 ^2^	0.95
2-NT	−0.92	-	-	-	−0.02	0.60 ^2^	-	-	-	-
3-NT	−0.84	-	-	-	−0.03	0.60 ^2^	-	-	-	-
RDX	−1.14	-	-	-	0.80	-	-	-	-	-
NG	−1.12 ^4^	-	-	-	0.78	-	-	-	-	-

^1.^ CSW parameters: Electrolyte = 50 mM potassium phosphate buffered at pH 6.5, frequency = 17.5 Hz, amplitude = 25 mV. Accumulation period =1 min at 1.2 and −1.8 V vs. Ag/AgCl, Current range =2 mA. ^2.^ Minor peak with the values italicized in the table. ^3.^ Shoulder, ^4.^ Broad peak.

## References

[1] Giannoukos S., Brkic B., Taylor S., Marshall A., Verbeck G.F. (2016). Chemical sniffing instrumentation for security applications. Chem. Rev..

[2] Moore D.S. (2004). Instrumentation for trace detection of high explosives. Rev. Sci. Instrum..

[3] Woodfin R.L. (2007). Trace Chemical Sensing of Explosives.

[4] Caygill J.S., Davis F., Higson S.P.J. (2012). Current trends in explosive detection techniques. Talanta.

[5] Trammell S.A., Hernandez S.C., Myers-Ward R.L., Zabetakis D., Stenger D.A., Gaskill D.K., Walton S.G. (2016). Plasma-modified, epitaxial fabricated graphene on sic for the electrochemical detection of tnt. Sensors.

[6] Parveen, Kant R. (2016). General theory for pulse voltammetric techniques on rough and finite fractal electrodes for reversible redox system with unequal diffusivities. Electrochim. Acta.

[7] Ryan P., Zabetakis D., Stenger D.A., Trammell S.A. (2015). Integrating paper chromatography with electrochemical detection for the trace analysis of tnt in soil. Sensors.

[8] Mohan A.M.V., Brunetti B., Bulbarello A., Wang J. (2015). Electrochemical signatures of multivitamin mixtures. Analyst.

[9] Trammell S.A., Zabetakis D., Moore M., Verbarg J., Stenger D.A. (2014). Square wave voltammetry of tnt at gold electrodes modified with self-assembled monolayers containing aromatic structures. PLoS ONE.

[10] Munir A., Shah A., Shah A.H., Rana U.A., Adhikari B., Khan S.B., Qureshi R., Kraatz H.B. (2014). Detailed electrochemistry of the environmental toxin ethylene diamine. J. Electrochem. Soc..

[11] Uzer A., Saglam S., Tekdemir Y., Ustamehmetoglu B., Sezer E., Ercag E., Apak R. (2013). Determination of nitroaromatic and nitramine type energetic materials in synthetic and real mixtures by cyclic voltammetry. Talanta.

[12] O’Mahony A.M., Wang J. (2013). Nanomaterial-based electrochemical detection of explosives: A review of recent developments. Anal. Methods.

[13] Ceto X., O’Mahony A.M., Wang J., del Valle M. (2013). Simultaneous identification and quantification of nitro-containing explosives by advanced chemometric data treatment of cyclic voltammetry at screen-printed electrodes. Talanta.

[14] Barry S., Dawson K., Correa E., Goodacre R., O’Riordan A. (2013). Highly sensitive detection of nitroaromatic explosives at discrete nanowire arrays. Faraday Discuss..

[15] Vuki M., Shiu K.K., Galik M., O’Mahony A.M., Wang J. (2012). Simultaneous electrochemical measurement of metal and organic propellant constituents of gunshot residues. Analyst.

[16] Galik M., O’Mahony A.M., Wang J. (2011). Cyclic and square-wave voltammetric signatures of nitro-containing explosives. Electroanalysis.

[17] Bandodkar A.J., O’Mahony A.M., Ramirez J., Samek I.A., Anderson S.M., Windmiller J.R., Wang J. (2013). Solid-state forensic finger sensor for integrated sampling and detection of gunshot residue and explosives: Towards ‘lab-on-a-finger’. Analyst.

[18] Yu H.A., Lee J., Lewis S.W., Silvester D.S. (2017). Detection of 2,4,6-trinitrotoluene using a miniaturized, disposable electrochemical sensor with an ionic liquid gel-polymer electrolyte film. Anal. Chem..

[19] Ceto X., O’Mahony A.M., Samek I.A., Windmiller J.R., del Valle M., Wang J. (2012). Rapid field identification of subjects involved in firearm-related crimes based on electroanalysis coupled with advanced chemometric data treatment. Anal. Chem..

[20] Gonzalez-Calabuig A., Ceto X., Del Valle M. (2016). Electronic tongue for nitro and peroxide explosive sensing. Talanta.

[21] Steinberg M.D., Lowe C.R. (2004). A micropower amperometric potentiostat. Sens. Actuators B: Chem..

[22] Cruz A.F., Norena N., Kaushik A., Bhansali S. (2014). A low-cost miniaturized potentiostat for point-of-care diagnosis. Biosens. Bioelectron..

[23] Friedman E.S., Rosenbaum M.A., Lee A.W., Lipson D.A., Land B.R., Angenent L.T. (2012). A cost-effective and field-ready potentiostat that poises subsurface electrodes to monitor bacterial respiration. Biosens. Bioelectron..

[24] Beach R.D., Conlan R.W., Godwin M.C., Moussy F. (2005). Towards a miniature implantable in vivo telemetry monitoring system dynamically configurable as a potentiostat or galvanostat for two- and three-electrode biosensors. IEEE Trans. Instrum. Meas..

[25] Stanacevic M., Murari K., Rege A., Cauwenberghs G., Thakor N.V. (2007). Vlsi potentiostat array with oversampling gain modulation for wide-range neurotransmitter sensing. IEEE Trans. Biomed. Circuits Syst..

[26] Rowe A.A., Bonham A.J., White R.J., Zimmer M.P., Yadgar R.J., Hobza T.M., Honea J.W., Ben-Yaacov I., Plaxco K.W. (2011). CheapStat: An open-source, “do-it-yourself” potentiostat for analytical and educational applications. PLoS ONE.

[27] Bard A.J., Faulkner L.R. (2001). Electrochemical Methods: Fundamentals and Applications.

[28] Billo E.J. (2011). Excel for Chemists A Comprehensive Guide.

[29] Lowry S.R. (2006). Automated spectral searching in infrared, raman and near-infrared spectroscopy. Handbook of Vibrational Spectroscopy.

